# Destructive inflammatory reaction after an autologous retinal pigment epithelium and choroid transplantation: no detection of an auto-immune response

**DOI:** 10.1186/s12348-022-00305-2

**Published:** 2022-08-26

**Authors:** Saskia H. M. van Romunde, Daphne P. C. Vergouwen, Daniela Iacovello, Dave L. Roelen, Robert M. Verdijk, Josianne C. E. M. ten Berge, Grazia Pertile, Marco W. J. Schreurs, Jan C. van Meurs

**Affiliations:** 1 Department of Vitreoretinal surgery, Rotterdam Eye Hospital, Schiedamse Vest 180 – 3011BH, Rotterdam, the Netherlands; 2 Department of Ophthalmology, Erasmus MC, University Medical Center Rotterdam, Doctor Molewaterplein 40, 3015 GD Rotterdam, the Netherlands; 3 Department of Immunology, Erasmus MC, University Medical Center Rotterdam, Doctor Molewaterplein 40, 3015 GD Rotterdam, the Netherlands; 4 Department of Ophthalmology, IRCCS Sacro Cuore Don Calabria, Viale Rizzardi, 437024 Negrar, Italy; 5 Department of Immunology, Leiden Universitary Medical Center, Albinusdreef 2, 2333 ZA Leiden, the Netherlands; 6 Department of Pathology, Section Ophthalmic Pathology, Erasmus MC, University Medical Center, Doctor Molewaterplein 40, 3015 GD Rotterdam, the Netherlands; 7 Department of Pathology, Leiden Universitary Medical Center, Albinusdreef 2, 2333 ZA Leiden, the Netherlands

**Keywords:** Age-related macular degeneration, Antibodies, Western blot, Immunofluorescence, Submacular surgery

## Abstract

**Purpose:**

Five patients who underwent uncomplicated retinal pigment epithelium (RPE)-choroid transplantation for neovascular age-related macular degeneration developed a destructive inflammatory reaction causing subretinal fluid accumulation and extensive RPE atrophy in the graft. We hypothesized that this inflammation could be caused by an auto-immune response against the graft, resulting in circulating auto-antibodies. The aim of our study was to examine a potential autoimmune origin, which would allow a more targeted therapy approach.

**Methods:**

Five above-mentioned patients and four control groups of five patients each were included: 1) after uncomplicated RPE-choroid transplantation, 2) after full macular translocation, 3) treated with anti-vascular endothelial growth factor, and 4) healthy controls. Histopathology of rejected graft tissue was performed using standard procedures. Presence of RPE-choroid autoantibodies in serum was examined by indirect immunofluorescence and Western blot, and human leukocyte antigen (HLA) typing was performed.

**Results:**

Histopathological examination of an explanted graft showed infiltration of T-lymphocytes and macrophages in the choroid and RPE, and an increased number of B-cell lymphocytes were found in the choroid. Indirect immunofluorescence showed weak RPE-choroid autoantibody immunoreactivity in three patients of different groups. Western blot did not show specific RPE-choroid autoantibody immunoreactivity and no difference of HLA genotypes between the groups was found.

**Conclusions:**

Although local mononuclear inflammatory cell infiltration and a high number of B-lymphocytes were observed in an explanted graft, we did not detect serological evidence of an autoimmune origin of the postoperative inflammation using direct immunofluorescence and Western Blot. Alternatively, the graft failure may have been caused by local innate inflammation, triggered by breakdown of tolerance. Based on our current findings of this small study group, we have no rationale to pursue therapies targeted towards autoreactive graft failure. More research is needed to confirm our findings.

**Supplementary Information:**

The online version contains supplementary material available at 10.1186/s12348-022-00305-2.

## Introduction

Neovascular age-related macular degeneration (AMD) is a common eye disease in the elderly population that is usually treated with intraocular injections of anti-vascular endothelial growth factor (anti-VEGF) [1]. Sometimes anti-VEGF is less effective, for example in case of a retinal pigment epithelial (RPE) tear, a large subretinal hemorrhage, or subretinal fibrosis. In these patients, surgery can be an alternative treatment option. In RPE-choroid transplantation and full macular translocation (FMT), the choroidal neovascularization (CNV) is surgically removed and the macula is provided with extrafoveal RPE in two different ways. Specifically, in RPE-choroid transplantation, a sheet of RPE and choriocapillaris is transplanted from the midperiphery to the macula, while in FMT the fovea is rotated to an extrafoveal area after counterrotation of the globe [2, 3].

In the Rotterdam Eye Hospital (REH) and Ospedale Sacro Cuore (OSC) RPE-choroid transplantation for AMD has been performed in more than 400 patients [3, 4]. An unusual postoperative course occurred several months after uncomplicated surgery in five patients, which was characterized by accumulation of subretinal fluid (SRF), development of hyperreflective spots (HRS) and diffuse RPE atrophy, suggesting a destructive inflammatory reaction.

The eye is unique in its ways to suppress inflammation. Several passive and active systems include the blood-retinal barrier, production of immunomodulatory cytokines, and suppression by regulatory T-cells [5]. Possibly, these systems were disrupted due to surgical stress or ischemic injury. According to the ‘danger theory’, distressed or injured cells can evoke an inflammatory response via damage-associated molecular patterns (DAMPs), activating a T-cell response [6]. We hypothesize that in the patients with the indicated inflammatory reaction after surgery, the transplanted RPE cells were recognized as ‘dangerous’ by antigen presenting cells, leading to the activation of an adaptive autoreactive T-cell response, and subsequent a B-cell mediated autoimmune response, illustrated by production of autoantibodies and eventually degradation of the graft.

Autoimmunity in several diseases is associated with certain human leukocyte antigen (HLA) genotypes, for example HLA-DR15 and multiple sclerosis [7]. Similarly, there could be an HLA predisposition in patients with the postoperative inflammation after RPE-choroid transplantation when its origin would be autoimmune.

The aim of this study was to investigate the potential role of an adaptive autoimmune response in the five patients that developed the inflammatory reaction after RPE-choroid transplantation, by identifying the presence of RPE-choroid reactive autoantibodies and evaluating a possible HLA-based genetic predisposition. An auto-immune mechanism would allow us to target specific factors in the autoimmune response, e.g. with monoclonal antibody therapy.

## Main text

### Materials and methods

This case-control study was conducted according to the Declaration of Helsinki and the Good Clinical Practice guidelines. The study was approved by the local ethical committee (trial number: NL72704.078.20).

#### Patients

Five patients that developed SRF, HRS, and diffuse RPE atrophy after uncomplicated RPE-choroid transplantation were included at the REH (Rotterdam, the Netherlands), or OSC (Negrar (Verona), Italy). Considering that presence of auto-antibodies has been described previously in AMD patients and even after laser photocoagulation [8, 9] four control groups were included each consisting of five patients: 1) patients that underwent uncomplicated RPE-choroid transplantation, 2) patients that underwent uncomplicated FMT, 3) AMD patients treated with anti-VEGF injections, and 4) healthy age-matched controls. Exclusion criteria were a history of uveitis, diabetic retinopathy, and a systemic autoimmune disease.

#### Data collection

Retrospectively, the following data were collected from medical files of the included patients with the inflammatory reaction: demographics, ophthalmic history, general history, indication for surgery, intra- and postoperative complications, and best-corrected visual acuity (BCVA) before surgery, after surgery and at last visit. Spectral Domain – optical coherence tomography (SD-OCT) images, fundus photography, fluoresceine angiography images and fundus autofluorescence (FAF) were collected. From the subjects of the control groups, demographics, ocular history and general history were reported. After informed consent was obtained, peripheral blood was collected providing both serum, and mononuclear cell samples for laboratory tests. The samples were stored at a temperature of − 80 °C until analyses.

#### Histopathology

In one patient the RPE-choroid graft was explanted because of a recurrent submacular hemorrhage and histopathologic examination was performed. The choroidal explant tissue was fixated in neutral buffered-formalin 10% and after processing, the formalin fixed paraffin embedded block was cut at 4 μm sections which were mounted on slides for histochemical and immunohistochemical staining. Routine staining protocols were used for standard haematoxylin and eosin staining using an automated staining system (HE600, Ventana Medical Systems, Tucsen, AZ, USA). Immunohistochemistry was performed with an automated, validated and accredited staining system (Ventana Benchmark ULTRA, Ventana Medical Systems, Tucsen, AZ, USA) using ultraview universal DAB detection kit. In brief, following deparaffinisation and heat-induced antigen retrieval, the tissue samples were incubated according to their optimized time with the antibody of interest. Incubation was followed by haematoxylin II counter stain for 8 minutes and then a blue coloring reagent for 8 minutes according to the manufacturer’s instructions (Ventana). Tonsil tissue was used as a positive control for all antibodies.

#### Indirect immunofluorescence

Serum anti-RPE-choroid antibodies and anti-retinal antibodies were determined as described previously, with slight optimization of the protocol [10]. In short, biochip primate retinal slides (Euroimmun, Lübeck, Germany) were incubated with patients’ serum for 30 minutes at room temperature (RT) (dilution 1:100). After incubation, the slides were washed with phosphate-buffered saline (PBS) for 15 minutes and incubated with goat anti-human IgG Cy5 (ab97172; Abcam, Cambridge, UK) for 30 minutes at RT. Tissue slides were washed again with PBS for 15 minutes, embedded in glycerol and covered with a coverslip. Slides were analysed and photographed using an Axioplan2 fluorescence microscope with AxioCam MRm at 160x magnification and an exposure time of 6 seconds (Zeiss, Thornwood, NY, USA). In case of a weak result the procedure was repeated with a serum dilution 1:50 and 1:25. PBS and serum negative for anti-nuclear antibodies (ANA) were used as negative references. Recombinant rabbit anti-RPE65 antibody (ab231782; dilution 1:250; Abcam, Cambridge, UK), recombinant anti-collagen type 1 (ab138492; dilution 1:500; Abcam, Cambridge, UK), and serum of an anti-retinal antibody positive, and ANA-positive subject (dilution 1:100) were used as positive references.

#### Western blot protein extracts

Human RPE and choroid tissue extracts for Western blot were prepared as described previously, implementing slight changes in the protocol [11]. RPE and choroid were obtained from a healthy donor eye post-mortem. After careful removal of the retina, RPE and choroid were dissected and subsequently homogenized in RIPA buffer with Halt protease inhibitor cocktail (Thermo Fisher Scientific, Waltham, USA) for 30 minutes. After homogenization the samples were centrifuged at 17000G at 4 °C for 15 minutes, the supernatants were separated, and protein yield was assessed by colorimetric Bradford assay. In addition to the human donor RPE-choroid protein extract, an RPE cell line was used to provide an RPE protein extract [12]. ARPE-19 cells were cultured in culture flasks in DME/F (HyClone, Logan, UT) containing 10% fetal calf serum, and 1% penicillin/streptomycin. Two times (72 h and 24 h before the harvest) 50 μg/ml ascorbic acid and 200 μM L-Proline were added to the cell culture, and thrombin (5 U/ml) was added 48 h before the harvest. Confluent grown ARPE19 cells were harvested and subsequently homogenized in cold RIPA buffer with Halt protease inhibitor cocktail (Thermo Fisher Scientific, Waltham, USA). After homogenization, the samples were centrifuged at 17000G at 4 °C for 15 minutes, the supernatants were separated, and protein yield was assessed by colorimetric Bradford assay. Extracts were stored at a temperature of − 80 °C until further use.

#### Western blot procedure

Protein extract were diluted in RIPA buffer, with 4x Laemmli sample buffer (Bio-rad, Hercules, California, USA), and incubated at 95 °C for 5 minutes. The lysate was loaded on 4–12% mini-PROTEAN TGX Stain-Free Precast Gels (Bio-rad, Hercules, California, USA), and transferred to nitrocellulose membranes. Membranes were blocked with trix-buffered saline (TBS) containing 3% bovine serum albumine (BSA), and subsequently incubated with diluted samples (serum 1:100 TBS-Tween 0,1% containing 3% BSA, other antibodies as mentioned) for 1 hour at RT. Membranes were washed multiple times using an automated laboratory workstation (auto-LIA 48; Fujirebio, Japan), and incubated with goat anti-rabbit IgG (IRDye 680; dilution 1:4000; Licor, Lincoln, NE, USA), or goat anti-Human IgG (IRDye 680; dilution 1:15000; Licor, Lincoln, NE, USA) for 30 minutes. Visualization was performed with the imaging system Odyssey CLx (Thermo Fisher Scientific, Waltham, USA). Recombinant rabbit anti-RPE65 antibody, anti-recoverin antibody, and recombinant anti-collagen type I antibody were used as references.

#### Human leukocyte antigen typing

DNA was collected from whole blood using a QIAamp DNA Blood Mini kit, or using PAXgene blood tubes and a PAXgene Blood DNA Kit (Qiagen, Hilden, Germany), following manufacturer protocol. HLA class I and II typing was performed on all samples except for the five healthy participants. The procedure was performed as described by van Sonderen et al. [13] The HLA type of the study subjects was compared to the frequency in 5604 Dutch healthy blood donors [13].

#### Statistical analyses

Because of the small number of patients, and the explorative nature of the study, no statistical analysis was performed. The data have been evaluated descriptively.

## Results

**Fig. 1:**
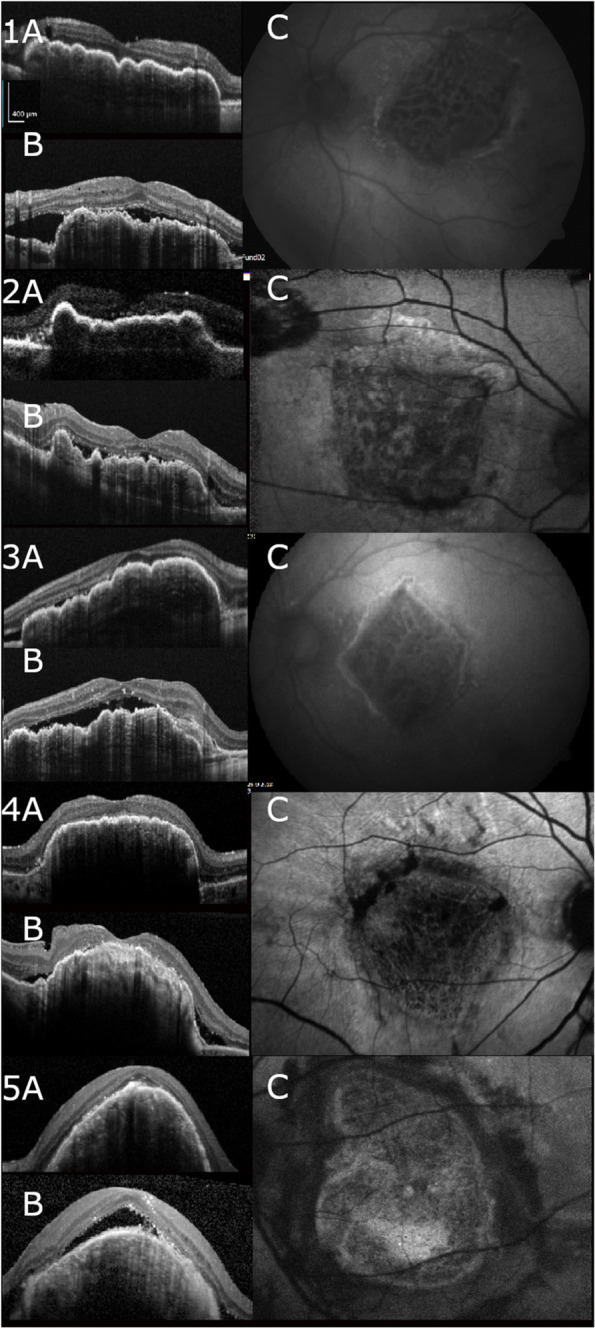
Imaging of patients with inflammatory reaction after RPE-choroid transplantation. Patient 1–5 after retinal pigment epithelium (RPE) – choroid transplantation. **A** Optical coherence tomography (OCT)-image taken 2–6 weeks after surgery show an intact layer of transplanted RPE. **B** OCT-image during inflammation showing subretinal fluid and diffuse atrophy of the transplanted RPE. **C** Fundus autofluorescence during inflammation illustrating a leopard-like pattern of atrophy

**Fig. 2:**
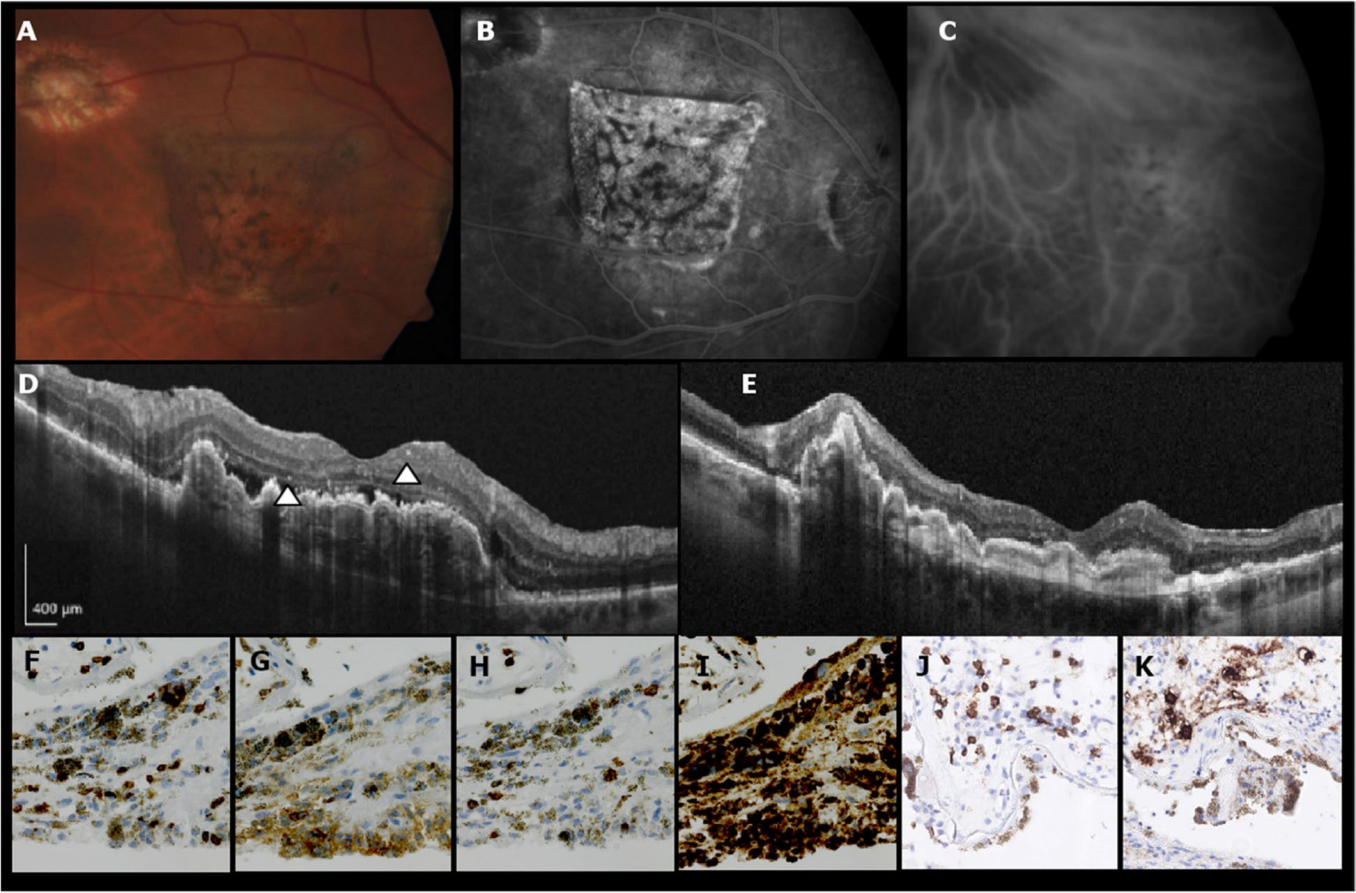
Imaging and histopathologic findings in a patient with an inflammatory reaction after RPE-choroid transplantation. Imaging (**A-E**) and histopathologic examination (**F-I**) of patient 2. Fundus shows a leopard-like pattern on the graft (**A**). At the time of inflammation fluorangiography shows no recurrence (**B**) and indocyanine green angiography shows perfusion of the graft (**C**). OCT-image during inflammation shows subretinal fluid and hyperreflective spots (*arrow heads*) (**D**). No inflammatory reaction occurred in the second RPE-choroid transplant 21 months after re-transplantation (**E**). Histopathology of the explanted graft demonstrates presence of CD3 positive T-lymphocytes (**F**), showing both CD4 positive T-helper phenotype (**G**), and CD8 positive cytotoxic T cell phenotype (**H**), and CD68 positive macrophages (**I**) in the choroid and RPE. An increased number of CD20 positive B-cells (**J**), of which a few were CD138 positive plasma cells (**K**), was seen in the choroid but not in the RPE

The demographics and ocular history of the five patients that developed an inflammatory reaction after RPE-choroid transplantation are shown in Table 1. None of the patients had a history of uveitis, or systemic autoimmune disease. Patient 1–3 were operated at REH and patient 4 and 5 were operated at OSC. On ophthalmic examination, there were no cells in the anterior or posterior segment. The onset of the inflammatory reaction after surgery ranged from 2 to 8 months. Figure 1 shows the changes of the OCT image, including SRF, HRS, and diffuse RPE atrophy. The latter is also clearly illustrated on FAF. Patient 2 developed a recurrent CNV with a large hemorrhage 2 years after primary surgery (Fig. 2). The graft was explanted and a second autologous graft was placed under the fovea. Interestingly, with a follow up period of 3 years, the inflammatory reaction did not recur in the new graft.

**Table 1:** Characteristics of patients with inflammatory reaction after retinal pigment epithelial (RPE)-choroid transplantation

Pt No.	Sex	Age	Eye	Ocular history	Indication for surgery	Onset^a^	BCVA preop	BCVA postop	BCVA inflammation	Blood withdrawal^b^
1	M	92	OS	None	RPE tear	3	20/200	20/40	20/63	15 & 42
2	F	84	OD	Submacular hemorrhage treated with vitrectomy, gas and r-tPA	RPE tear	8	20/125	20/125	20/200	13 & 37
3	F	86	OS	Glaucoma, phaco	RPE tear	5	20/100	20/100	20/200	0 & 24
4	M	71	OD	Glaucoma treated with Baerveldt implant, anti-VEGF	Fibrotic scar	2	20/40	20/40	20/1250	106
5	F	73	OS	Anti-VEGF	Fibrotic scar	3	20/100	20/40	20/80	43

^a^Onset of inflammatory reaction after RPE-choroid transplantation in months

^b^Time between inflammation and blood withdrawal in months

*Anti-VEGF* Anti-vascular endothelial growth factor, *BCVA* Best-corrected visual acuity, *RPE* Retinal pigment epithelium, *r-tPA* Recombinant tissue plasminogen activator

Histopathologic assessment of the explanted first graft showed mononuclear inflammatory cell infiltration of both helper- and cytotoxic T-lymphocytes and macrophages in the RPE and choroid, based on increased presence of cells expressing CD3, CD4, CD8 and CD68, respectively. An increased number of B-lymphocytes, of which a few were plasma cells, were seen only in the choroid but not in the RPE, based on the presence of cells expressing CD20 and CD138, respectively. Patient 1–3 were treated with peribulbar steroid injection and patient 4 and 5 with topical steroids after the inflammatory reaction. In all patients SRF resolved after several months. The blood samples were withdrawn 0–106 months after the inflammatory reaction. In patient 1–3 a second sample was taken 24–42 months after the inflammation.

Demographics of the control groups are shown in Table 2. The groups RPE-choroid transplantation, anti-VEGF injection, and healthy participants were recruited at the REH and patients that underwent FMT were recruited at OSC.

**Table 2:** Characteristics of the control groups

	RPE-choroid transplantation (*n* = 5)	Full macular translocation (*n* = 5)	Anti-VEGF injection (*n* = 5)	Healthy participants (*n* = 5)
Male, no (%)	3 (60%)	3 (60%)	3 (60%)	3 (60%)
Mean age (SD, range)	71 (5, 65–78)	82 (3, 77–84)	84 (5, 78–90)	72 (4, 66–77)

*Anti-VEGF* Anti-vascular endothelial growth factor, *RPE* Retinal pigment epithelium, *SD* Standard deviation

### Autoantibody detection by indirect immunofluorescence

**Fig. 3:**
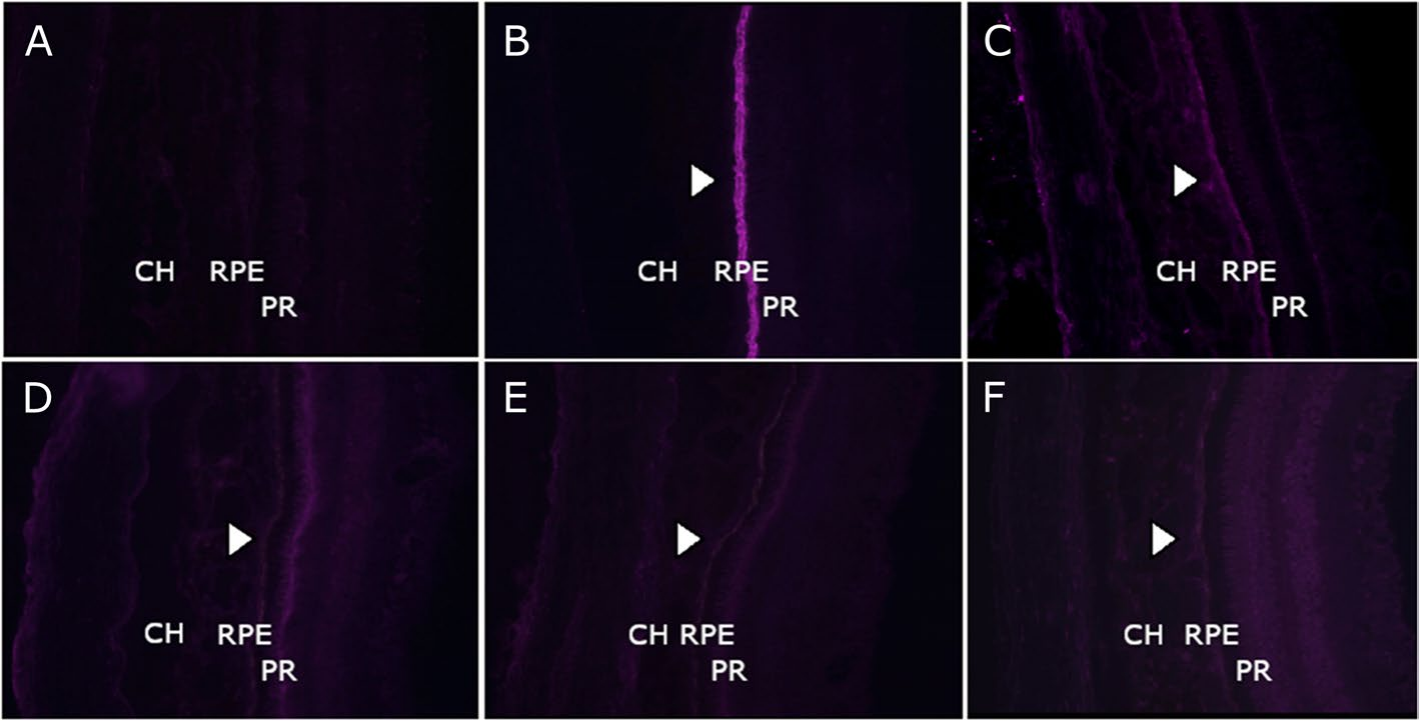
Indirect immunofluorescence. Indirect immunofluorescence of references (**A-C**) and serum of patients (dilution 1:100) with weak fluorescence of the retinal pigment epithelium (RPE) and photoreceptors (*arrow heads*) (**D-F**). Photographs were taken at 160x magnification and 6 seconds exposure time. **A** healthy control. **B** Recombinant rabbit anti-RPE65 antibody. **C** anti-recoverin antibody. **D** patient with inflammation after RPE-choroid transplantation. **E** patient that underwent full macular translocation. **F** patient after uncomplicated RPE-choroid transplantation. CH = choroid, PR = photoreceptor, RPE = retinal pigment epithelium

Negative controls, positive anti-RPE65 and anti-retinal antibodies confirmed the validity of autoantibody detection by indirect immunofluorescence of ocular tissue (Fig. 3A-C). There was no strong immunoreactivity against RPE-choroid or other ocular structures observed in the serum of any of the subjects. In three patients there was weak fluorescence of the RPE and the photoreceptors: patient number 4 of the inflammation group, one patient in the FMT group, and one patient in the uncomplicated RPE-choroid transplantation group (Fig. 3D-F). The fluorescence of the RPE increased with higher serum concentrations (1:50 and 1:25), but the signal remained weak. In one FMT patient, one patient after uncomplicated RPE-choroid transplantation and two patients treated with anti-VEGF only, various intraretinal structures (photoreceptor layer, ganglion cell layer, inner plexiform layer) showed weak fluorescence with increasing intensity with higher serum concentrations. The presence of antibodies in both disease and control groups indicates a lack of specific autoantibody immunoreactivity.

### Autoantibody detection by Western blot

**Fig. 4:**
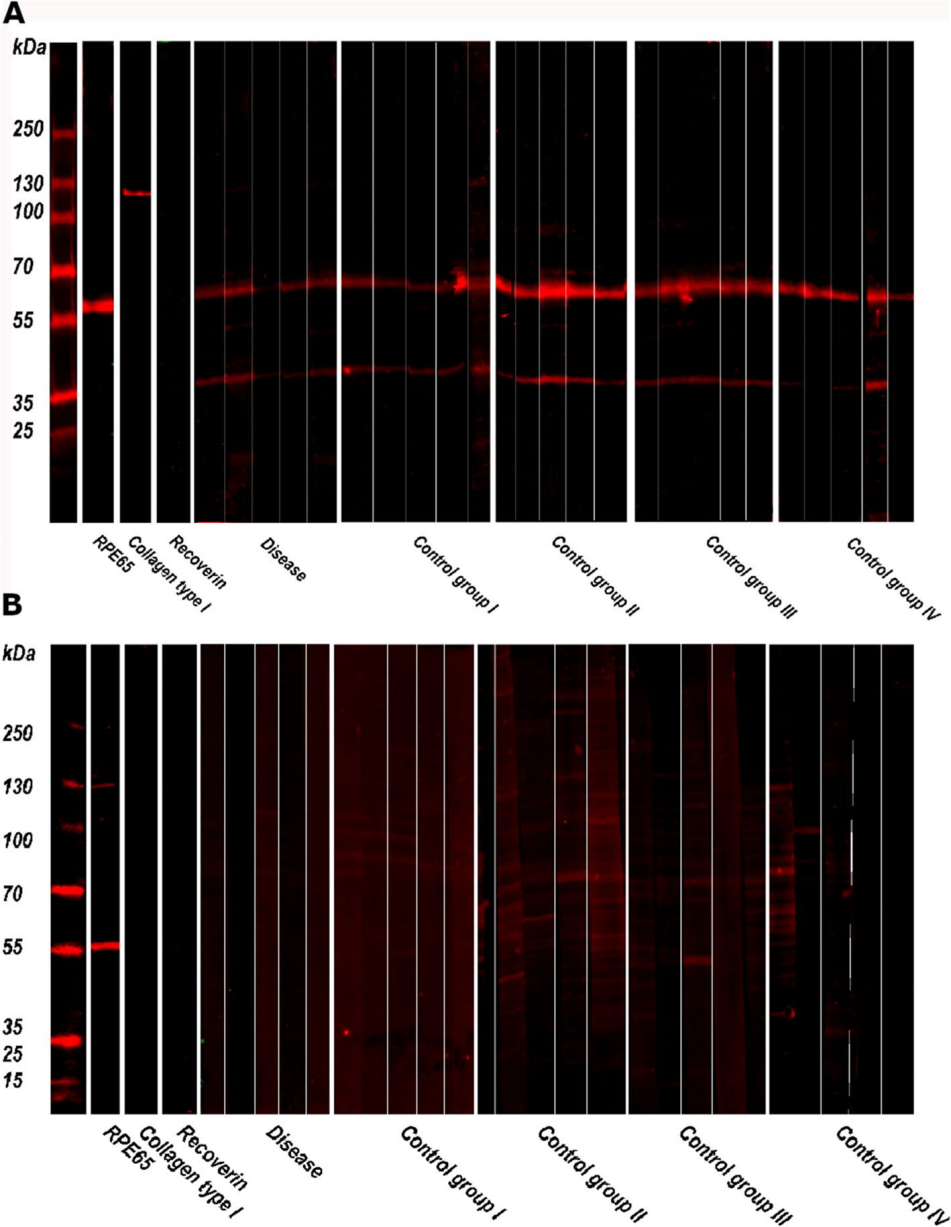
Western blots. **A**: Western blot using retinal pigment epithelium (RPE)- and choroid lysate. RPE-65 and collagen type 1 show a immunoreactive band. Beside background staining, there is no reactivity in any of the patients. **B**: Western blot using protein extract of an RPE cell line. RPE-65 showed immunoreactive staining. There is no staining in patients that developed an inflammatory reaction after RPE-choroid transplantation (*Disease*), and nonspecific weak staining in patients that underwent uncomplicated RPE-choroid transplantation (*Control group I*), patients that underwent full macular translocation (*Control group II*), age-related macular degeneration patients treated with anti-vascular endothelial growth factor (*Control group III*), and healthy controls (*Control group IV*)

The results of the Western blots performed using the donor RPE-choroid tissue lysate and protein extract of the RPE-cell line are illustrated in Fig. 4. In both Western blots anti-RPE65 was positive and anti-recoverin was negative, validating the protein content of the lysates. Anti-collagen type 1 was positive with high intensity light exposure in the Western blot using donor tissue lysate, indicating the presence of choroidal tissue. This Western blot showed complete absence of potential autoantibody immunoreactivity in all patients. The positive signals at ~ 40 kDa and ~ 60 kDa could be explained by the presence of immunoglobulins in human donor RPE-choroid tissue. The Western blot using the RPE cell line demonstrated no evident immunoreactivity in the inflammation group as well. There were several weak signals mostly in the group of uncomplicated RPE-choroid transplantation. For the most part these signals did not seem to be specific. The patients with weak immunoreactivity on Western blot did not match those patients with weak immunoreactivity on indirect immunofluorescence, indicating lack of relevance of these observations.

### HLA typing

There was no overrepresentation of an HLA class I or class II type in patients that developed the inflammatory reaction as compared to the included controls and to the general population. No significant differences could be found between the inflammation group, the control groups, and the healthy subjects. See Supplementary Table S1 for complete results.

## Discussion

The aim of our study was to examine a potential autoimmune origin of RPE-choroid transplant failure. Our initial hypothesis is supported by the presence of a mononuclear inflammatory infiltrate, including T-cells, in the failed graft and a high number of B-lymphocytes in the choroid. No serological evidence, however, of a systemic humoral response was detected using indirect immunofluorescence as well as Western blot analyses in patients who developed an inflammatory reaction after uncomplicated RPE-choroid transplantation. In addition, we were unable to identify a specific HLA association as possible genetic predisposition for autoimmune mediated graft failure.

The role of humoral autoimmunity in graft failure, activated by T-helper cells, has become more clear in recent years [14]. Presence of autoantibodies has been found after allogeneic and autologous transplantation [14–17]. Along the same line, we hypothesized that the inflammatory reaction was associated with an adaptive response of autoimmune origin resulting in the formation of detectable autoantibodies against RPE-choroid tissue and overrepresentation of a certain HLA type. The results of our study, however, do not support our theory.

The appearance of the graft and findings on multimodal imaging, i.e. SRF, HRS, and RPE atrophy, implies a destructive inflammatory process. It is common to notice a thin layer of SRF underneath the graft, which disappears when revascularisation is established [18]. However, in the presented patients the fluid appeared in a later phase and persisted for several months. HRS are believed to represent migrated RPE cells or activated microglial cells [19]. The latter cells have an important role in the innate immune system, and possibly in the pathogenesis of several retinal diseases including AMD [20, 21]. Postoperative RPE atrophy in the graft is highly prevalent after RPE-choroid transplantation [3]. Normally it initiates at the edge of the graft and slowly expands towards the centre. Expansion is more rapid in areas with a large gap between the native RPE and transplanted RPE [22]. However, the sudden onset and the involvement of the entire graft suggest an inflammation instead of a degenerative process. Moreover, the histopathological examination of the explanted graft showed infiltration of mononuclear inflammatory cells.

There are several possible explanations for our results. The immune reaction may have been targeted specifically against injured RPE. According to the danger theory, DAMP molecules induce inflammation to eliminate the injured cells [6, 23]. In that case, we may not expect any adaptive immune reactivity against healthy RPE-choroid tissue. Although some antibody responses persist lifelong, others last only a short time [14]. Possibly, autoantibodies against the transplanted tissue were produced in a concentration too low to detect in serum or the serum was sampled too long after the actual autoantibody production. Concentrations may have been higher either locally at the inflammation site or earlier during the failure process. Unfortunately, we did not have any ocular fluids or tissues available to examine local deposition of autoantibodies. Alternatively, it is possible that only a local T-cell mediated inflammation was causing SRF and damage to the RPE of the graft. Studying autoreactive T-cells is highly challenging in this matter, therefore we first investigated the indirect consequence of an autoreactive response by T-cells, using detection of autoantibodies. Without further evidence for autoreactivity we believe it is not feasible to further study antigen-specific T-cells. Moreover, absence of inflammation after transplantation of a second graft in one patient implies that there would be no autoimmune origin. Still, it remains unclear what triggered the inflammation, since the surgery and initial postoperative course in all five patients were uncomplicated.

Detection of autoantibodies is generally used for diagnostic purposes when autoimmune retinopathy is suspected. In the last decade, the interest for ocular autoantibodies has broadened. There is growing evidence for the involvement of autoantibodies in the pathogenesis of other ocular diseases, including AMD [24]. It has been demonstrated that autoantibodies to macular tissue are significantly more prevalent and show stronger autoreactivity in AMD patients compared to age-matched controls [25]. Patel et al. found a higher titre of anti-retinal antibodies in dry and exudative AMD patients compared to controls (94%, 82% and 9%, respectively) [9]. In agreement with the literature, we noticed more immunofluorescent reactivity in the RPE layer and neuroretina in AMD patients compared to healthy controls in our study. Drusen containing complement factors and IgG immune complexes add to the suggestion that immune-related processes play a role in the pathogenesis of AMD [26]. The question whether such autoantibody-driven immunoreactivity is pathogenic or merely an epiphenomenon of tissue destruction is still largely unanswered. Further research into this topic is important because autoimmune biomarkers might be useful for diagnoses, prognoses and perhaps as a target for AMD treatment [24].

This study has several limitations. Evidently, the number of patients is too low to draw definitive conclusions. Autoantibody concentration may have been insufficient for detection due to the long period between onset of inflammation and blood sample collection in some patients. The validity of antiretinal antibody testing has been questioned previously because of false-positive binding [27]. Probably, the reported low validity may be influenced by lack of standardization of the laboratory assessment [10, 28]. In our study, all assessments were performed in one laboratory according to previously described protocols. The autoimmune origin of the observed inflammation could be primarily T-cell mediated. Unfortunately, we were unable to specifically examine anti-RPE-choroid T-cell immunoreactivity due to technical limitation. Although the low number of patients precludes any conclusion, the lack of HLA type association with the inflammatory reaction may argue against autoreactive T-cell involvement.

## Conclusion

Assessment with indirect immunofluorescence and Western blot of RPE-choroid reactive autoantibodies and HLA typing did not show evidence for an autoimmune origin of the inflammatory complication, in spite of the observed numerous B-cells in the choroid and the local tissue infiltration by T-cells. Therefore, in this series of patients, we found no rationale to pursue more specific and off-label targeted immunomodulatory therapies such as anti-B-cell antibodies. Possibly the inflammation was caused by a local innate inflammation only, triggered by a breakdown of tolerance and weakening of the blood-retinal barrier. Moreover, there are important drawbacks including a small sample size and limitations of the laboratory assessments. Although we were unable to pinpoint the cause in our series of patients, we consider it important to recognize and study this complication, and to establish a diagnostic set-up, as we may encounter this phenomenon more often in the future. Though submacular surgery for AMD is rarely performed since the introduction of anti-VEGF, there is a growing interest in the transplantation of induced pluripotent stem cells and embryonic stem cell derived RPE [29].

## Supplementary Information

**Additional file 1: Supplementary Table S1.** Human leukocyte antigen (HLA) results.

## Abbreviations

ANA: Antinuclear antibodies
Anti-VEGF: Anti-vascular endothelial growth factor
BCVA: Best-corrected visual acuity
BSA: Bovine serum albumine
CNV: Choroidal neovascularization
DAMP: Damage-associated molecular pattern
FAF: Fundus autofluorescence
FMT: Full macular translocation
HLA: Human leukocyte antigen
HRS: Hyperreflective spots
PBS: Phosphate-buffered saline
REH: Rotterdam Eye Hospital
RPE: Retinal pigment epithelium
RT: Room temperature
OSC: Hospital Sacro Cuore – Don Calabria
SD-OCT: Spectral-domain optical coherence tomography
SRF: Subretinal fluid
TBS: Trix-buffered Saline
