# Supplementary material for: Destructive inflammatory reaction after an autologous retinal pigment epithelium and choroid transplantation: no detection of an auto-immune response
Source: J Ophthalmic Inflamm Infect. 2022 Aug 26;12:27. doi: 10.1186/s12348-022-00305-2 (PMC9418395; doi:10.1186/s12348-022-00305-2)
Supplement: Supplementary file 1 — Additional file 1: Supplementary Table S1. Human leukocyte antigen (HLA) results. [file 12348_2022_305_MOESM1_ESM.docx]

**Supplementary table S1: Human leukocyte antigen (HLA) results**

| HLA type | Inflammation group % (n = 5) | Control groups % (n = 15) | Healthy donors % (n =5604) |
| --- | --- | --- | --- |
| A1 | 60 | 27 | 34 |
| A2 | 60 | 33 | 49 |
| A3 | 0 | 27 | 29 |
| A11 | 20 | 7 | 12 |
| A23 | 20 | 7 | 2 |
| A24 | 0 | 33 | 16 |
| A25 | 0 | 7 | 2 |
| A26 | 0 | 7 | 4 |
| A29 | 0 | 13 | 5 |
| A30 | 0 | 7 | 5 |
| A31 | 0 | 7 | 6 |
| A32 | 20 | 13 | 6 |
| A33 | 0 | 7 | 2 |
| A68 | 0 | 13 | 9 |
| B7 | 0 | 7 | 25 |
| B8 | 40 | 13 | 26 |
| B13 | 0 | 13 | 4 |
| B18 | 0 | 13 | 7 |
| B27 | 20 | 0 | 7 |
| B35 | 20 | 20 | 19 |
| B38 | 0 | 7 | 3 |
| B39 | 0 | 13 | 4 |
| B44 | 20 | 53 | 22 |
| B50 | 0 | 7 | 1 |
| B51 | 20 | 13 | 11 |
| B57 | 0 | 7 | 7 |
| B58 | 0 | 7 | 2 |
| B62 | 20 | 7 | - |
| B63 | 20 | 0 | 1 |
| B64 | 0 | 7 | 4 |
| Bw4 | 60 | 73 | - |
| Bw6 | 60 | 67 | - |
| Cw1 | 20 | 0 | 6 |
| Cw3 | 20 | 7 | 30 |
| Cw4 | 40 | 13 | 23 |
| Cw5 | 0 | 40 | 14 |
| Cw6 | 0 | 33 | 16 |
| Cw7 | 60 | 47 | 56 |
| Cw8 | 0 | 7 | 4 |
| Cw9 | 20 | 7 | - |
| C*12 | 0 | 20 | 8 |
| C*15 | 0 | 7 | 5 |
| C*16 | 20 | 13 | 6 |
| DR1 | 0 | 13 | 22 |
| DR4 | 40 | 20 | 28 |
| DR7 | 40 | 20 | 20 |
| DR9 | 0 | 7 | 3 |
| DR11 | 0 | 13 | 17 |
| DR12 | 0 | 7 | 4 |
| DR13 | 20 | 40 | 25 |
| DR14 | 40 | 7 | 7 |
| DR15 | 20 | 20 | 24 |
| DR16 | 0 | 7 | 3 |
| DR17 | 40 | 27 | 28 |
| DR51 | 20 | 27 | - |
| DR52 | 80 | 80 | - |
| DR53 | 60 | 80 | - |
| DQ2 | 80 | 47 | 39 |
| DQ5 | 40 | 27 | 33 |
| DQ6 | 20 | 40 | 44 |
| DQ7 | 0 | 33 | 30 |
| DQ8 | 40 | 13 | 19 |
| DQ9 | 0 | 7 | 9 |
